# Human IGF1 Regulates Midgut Oxidative Stress and Epithelial Homeostasis to Balance Lifespan and *Plasmodium falciparum* resistance in *Anopheles stephensi*

**DOI:** 10.1371/journal.ppat.1004231

**Published:** 2014-06-26

**Authors:** Anna L. Drexler, Jose E. Pietri, Nazzy Pakpour, Eric Hauck, Bo Wang, Elizabeth K. K. Glennon, Martha Georgis, Michael A. Riehle, Shirley Luckhart

**Affiliations:** 1 Department of Medical Microbiology and Immunology, School of Medicine, University of California, Davis, Davis, California, United States of America; 2 Department of Entomology, University of Arizona, Tucson, Arizona, United States of America; Stanford University, United States of America

## Abstract

Insulin and insulin-like growth factor signaling (IIS) regulates cell death, repair, autophagy, and renewal in response to stress, damage, and pathogen challenge. Therefore, IIS is fundamental to lifespan and disease resistance. Previously, we showed that insulin-like growth factor 1 (IGF1) within a physiologically relevant range (0.013–0.13 µM) in human blood reduced development of the human parasite *Plasmodium falciparum* in the Indian malaria mosquito *Anopheles stephensi*. Low IGF1 (0.013 µM) induced FOXO and p70S6K activation in the midgut and extended mosquito lifespan, whereas high IGF1 (0.13 µM) did not. In this study the physiological effects of low and high IGF1 were examined in detail to infer mechanisms for their dichotomous effects on mosquito resistance and lifespan. Following ingestion, low IGF1 induced phosphorylation of midgut c-Jun-N-terminal kinase (JNK), a critical regulator of epithelial homeostasis, but high IGF1 did not. Low and high IGF1 induced midgut mitochondrial reactive oxygen species (ROS) synthesis and nitric oxide (NO) synthase gene expression, responses which were necessary and sufficient to mediate IGF1 inhibition of *P. falciparum* development. However, increased ROS and apoptosis-associated caspase-3 activity returned to baseline levels following low IGF1 treatment, but were sustained with high IGF1 treatment and accompanied by aberrant expression of biomarkers for mitophagy, stem cell division and proliferation. Low IGF1-induced ROS are likely moderated by JNK-induced epithelial cytoprotection as well as p70S6K-mediated growth and inhibition of apoptosis over the lifetime of *A. stephensi* to facilitate midgut homeostasis and enhanced survivorship. Hence, mitochondrial integrity and homeostasis in the midgut, a key signaling center for IIS, can be targeted to coordinately optimize mosquito fitness and anti-pathogen resistance for improved control strategies for malaria and other vector-borne diseases.

## Author Summary

The complexity of the malaria parasite life cycle makes it an elusive target for drug and vaccine development. Thus, targeting the parasite in the mosquito vector is an attractive alternative. When consuming an infective blood meal the mosquito ingests not only the blood proteins and parasites, but a range of host blood factors, including the insulin-like growth factor-1 (IGF1) hormone. IGF1 is a highly conserved signaling molecule that regulates a broad spectrum of cellular processes, including immunity and midgut homeostasis. We previously demonstrated that human IGF1 ingested in a blood meal can induce cell signaling in the mosquito midgut that reduces malaria parasite development and extends mosquito lifespan. In this study, we show that midgut signaling by human IGF1 increased the synthesis of reactive oxygen species in midgut mitochondria and enhanced nitric oxide synthase gene expression, responses that inhibit malaria parasite development in the mosquito. Additionally, we found that IGF1 signaling facilitates midgut homeostasis to enhance mosquito survival. These results suggest that IGF1 signaling in the mosquito midgut could be targeted to coordinately enhance mosquito fitness and anti-parasite resistance for improved malaria control strategies.

## Introduction

More than fifty percent of the world's population is currently at risk for malaria infection. *Plasmodium falciparum*, the most virulent of the human malaria parasites, infects approximately 250 million people each year [1], a disproportionate number of whom are children under age five. Current control measures include drugs and insecticides, but increased prevalence of resistant parasite strains and mosquitoes [2] has necessitated the development of novel control strategies, including use of genetically modified mosquitoes, to combat this disease.

The definitive hosts for anthroponotic species of *Plasmodium* are mosquitoes in the genus *Anopheles*. Parasites undergo rapid developmental transitions in the midgut while mosquito immune barriers function coordinately to limit infection [3], [4]. Primary physical and chemical barriers of the midgut, including increased levels of reactive oxygen species (ROS) and nitric oxide (NO) kill parasites in the gut lumen, preventing contact between parasites and the epithelium [5]–[10]. Secondary defenses are activated when parasites encounter the midgut epithelium and include the production of nuclear factor (NF)-κB-dependent immune effectors such as antimicrobial peptides (AMPs) and complement-like factors which together function to further limit parasite development [3], [4]. Additionally, human hormones, cytokines, and growth factors that are ingested with the infectious blood meal can regulate mosquito physiology to affect parasite development [11]. For example, we have shown that the Indian malaria mosquito *Anopheles stephensi* can respond to ingested human insulin [12]–[14]. Insulin can rise to high levels in blood during malaria infection [15], [16] and, when ingested during mosquito feeding, activates canonical insulin/insulin-like growth factor signaling (IIS) in the midgut to reduce mosquito lifespan and increase *P. falciparum* infection [12]–[14]. *A. stephensi* can also respond to ingested human IGF1 [17], which unlike insulin, declines in serum during malaria [18]. In particular, levels of IGF1 in blood of healthy humans can fall from 0.09 µM [19], [20] to below 0.006 µM during severe infections with *P. falciparum* and *Plasmodium vivax* [18]. To understand the impact of ingested human IGF1 on *A. stephensi*, we examined the effects of 0.013 µM IGF1 (physiologically low) and 0.13 µM IGF1 (physiologically high) on mosquito lifespan and *P. falciparum* infection. Intriguingly, we noted that both concentrations provided in a blood meal reduced *P. falciparum* infection, while low IGF1 significantly extended lifespan relative to high IGF1-fed mosquitoes, which were not significantly different from controls [17].

The effects of insulin and IGF1 on IIS in *A. stephensi* are distinct and likely dictate the markedly different effects of these growth factors on lifespan and infection. In particular, human insulin induces phosphorylation of FOXO in the *A. stephensi* midgut [13] as does low IGF1 [17], whereas high IGF1 has no effect on FOXO [17]. Low IGF1 induces phosphorylation of p70S6K, a protein that is not activated by insulin, and both low and high IGF1 repress phosphorylation of ERK [17], a protein that is activated by insulin in the *A. stephensi* midgut [12], [13]. Given that IGF1 regulation of p70S6K is crucial to inhibition of BCL2-mediated apoptosis [21], we speculated that lifetime treatment of *A. stephensi* with a low concentration of IGF1 may optimally regulate apoptosis for midgut homeostasis, resulting in longer-lived mosquitoes.

A significant body of literature points to IIS regulation of epithelial homeostasis through a balance of death (apoptosis), survival (autophagy) and renewal (stem cell proliferation and differentiation) of epithelial cells to define longevity and disease resistance. For example, when the *D. melanogaster* midgut is damaged, enterocytes are extruded into the *D. melanogaster* gut lumen and display both increased caspase-3 activity, indicating an irreversible commitment to apoptotic cell death, and hallmarks of autophagy, including histological evidence of autophagosomes and enhanced gene expression of autophagy related gene 8 (ATG8) [22]. Autophagy can modulate lifespan and senescence [23] and several studies have shown connections among IIS, autophagy, and pathogen resistance as well. In *C. elegans*, pathogen resistance and lifespan extension are dependent on autophagy gene expression in IIS-inhibited Daf-2 mutants [24], [25]. A number of recent studies have also shown that IIS in *D. melanogaster* can alter lifespan through complex effects on gut epithelial renewal [26]. Long-lived IIS mutants are surprisingly deficient in proliferative responses to tissue damage [27], yet exhibit enhanced pathogen resistance [28], [29]. Moderate reduction in IIS in intestinal stem cells (ISCs), in contrast, can reduce cell proliferation and extend *Drosophila* lifespan, while inhibition of IIS in ISCs impaired midgut renewal and shortened lifespan, indicating that an *optimal* level of IIS is necessary to regulate gut epithelial proliferation and repair, lifespan, and host defense [30].

In *A. stephensi*, enhanced IIS via Akt overexpression in the midgut reduced expression of genes associated with autophagy (*ATG6*, *ATG8*) and epithelial renewal (*Esg*, *Pros*), resulting in the accumulation of dysfunctional mitochondria and toxic reactive nitrogen and oxygen species (RNOS) that damaged the midgut and shortened mosquito lifespan [31]. Conversely, when the IIS antagonist phosphatase and tensin homolog (PTEN) was overexpressed to repress IIS in the mosquito midgut, increased autophagy and enhanced expression of markers of epithelial renewal were observed in concert with extended lifespan and enhanced pathogen resistance [32]. From our own observations as well as studies of model organisms, we have inferred that IIS is a central regulator of gut epithelial homeostasis in *A. stephensi*. Further, this biology is mediated by changes in RNOS, mitochondrial integrity, and stem cell maintenance and differentiation, which can coordinately modulate lifespan and pathogen resistance [31]. Here, we sought to define IIS-dependent biological processes that define IGF1 phenotypes in *A. stephensi*. To this end, we examined outputs of NF-κB-dependent immunity in the context of key parameters of gut epithelial homeostasis in *A. stephensi* to define IGF1-specific features of longevity and parasite resistance.

## Results

### Human IGF1 induced JNK activation, but not NF-κB-dependent immune responses, in *A. stephensi*

**Figure 1:**
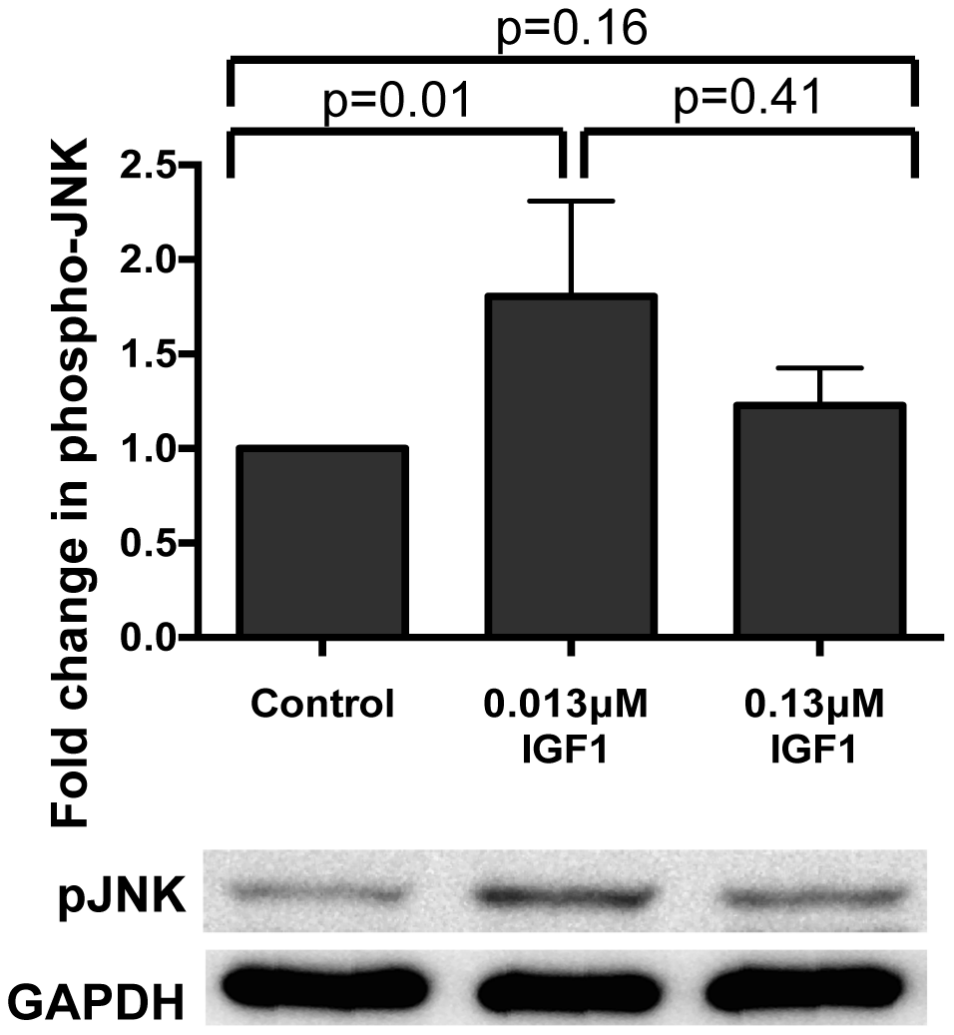
Ingested human IGF1 increased JNK phosphorylation in *A. stephensi* midguts. Mosquitoes were fed blood meals containing 0.013 µM or 0.133 µM IGF1 or an equivalent volume of PBS. Midguts were dissected at 0.5–1 h after the initiation of blood feeding. Data are represented as the average fold induction±SEM of phospho-JNK quantified by densitometry and normalized first to the GAPDH loading control and then to the buffer control. A representative western blot is shown below the graph. Data were analyzed by Mann Whitney test. Experiments were replicated four times and p-values are displayed on the graphs.

We have previously shown that ingested human insulin inhibits NF-κB-dependent immunity in *A. stephensi* leading to increased malaria parasite development [13], [14]. Therefore, we reasoned that ingested IGF1 might activate NF-κB-dependent immunity to inhibit *P. falciparum* development in the mosquito. In addition to IIS regulation, IGF1 can potently activate the mitogen-activated protein kinase (MAPK) c-Jun N-terminal kinase (JNK) via receptor-interacting protein (RIP) [33]. The RIP family proteins are crucial mediators for multiple inputs that activate NF-κB-dependent responses [34] and encode death domains orthologous to those of immune deficiency (imd) [35], a major regulator of NF-κB-dependent immunity in *D. melanogaster* [36] and *Anopheles* mosquitoes [37]. Given that *Plasmodium berghei* killing in *Anopheles gambiae* has been associated with JNK activation [38] and that JNK functions cooperatively with NF-κB mediated defenses downstream of TAK1 activation [39]–[42], we reasoned that IGF1 might activate *A. stephensi* JNK to upregulate NF-κB-dependent and/or imd-associated anti-parasite responses. We first assessed the effects of IGF1 treatment on midgut levels of JNK activation in *A. stephensi* using phospho-specific antisera and observed that only low concentrations of IGF1 significantly induced JNK activation in the *A. stephensi* midgut relative to controls at 30 min post-feeding (Figure 1). While relative induction levels of low and high IGF1 were not significantly different from one another, high IGF1 showed only a trend towards moderately increased JNK activation (20%) relative to controls (Figure 1).

**Figure 2:**
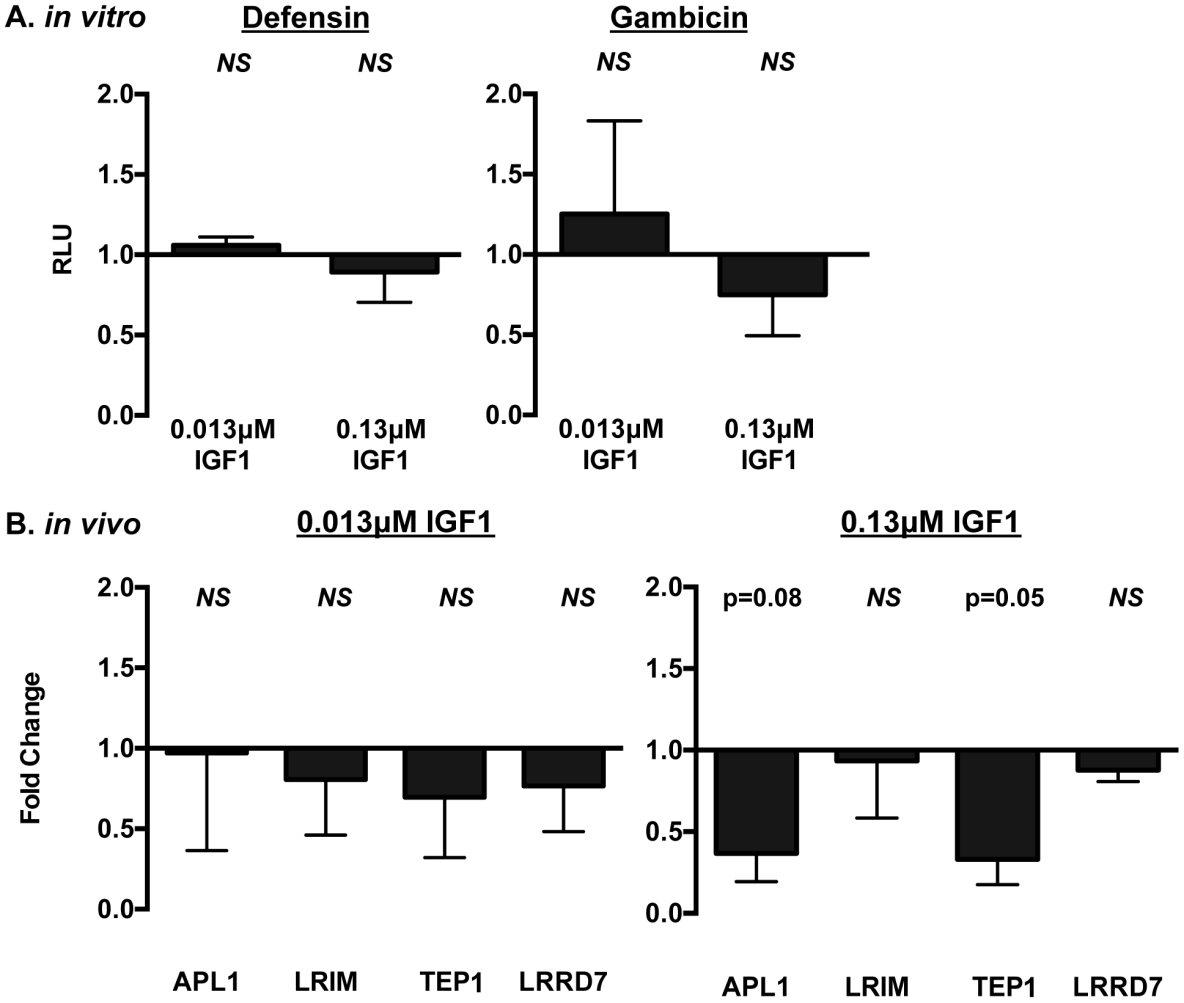
IGF1 did not induce prototypical NF-κB-dependent immune responses in *A. stephensi* cells *in vitro* (A) or *in vivo* (B). (A) Human IGF1 did not alter antimicrobial peptide (AMP) promoter activity in immune-activated ASE cells. Graphs depict fold change in relative light units (RLU) from promoter-reporter assays in transfected cells stimulated with or without LPS and with or without IGF1. Promoter activities following treatment were normalized first to levels in matched controls without LPS treatment, then to levels in matched controls treated with LPS and without IGF1 to define IGF1-specific changes in promoter activity. Data were analyzed by unpaired t-tests comparing each treatment to the matched buffer control; NS indicates p>0.1. Defensin n = 4; Gambicin n = 3. (B) Ingested human IGF1 reduced or had no effect on expression of immune effector genes APL1, TEP1, LRIM and LRRD7. Mosquitoes were fed artificial blood meals that were supplemented with *P. falciparum* FTPP and treated with 0.013 or 0.13 µM IGF1 or an equivalent volume of PBS as a control. Midguts were dissected 24 h after the initiation of blood feeding and RNA isolated for quantitative RT-PCR. Data are represented as fold change (2**^−ΔΔCt^**) in immune gene expression in FTPP-treated *A. stephensi* relative to matched control (PBS). Data were analyzed using Kruskal-Wallis non-parametric tests followed by Dunn's post-tests. Experiments were replicated six times with separate cohorts of mosquitoes and p-values are indicated.

We next examined the effects of IGF1 treatment on induction of NF-κB-dependent responses in *A. stephensi* cells *in vitro* and *in vivo*. For *in vitro* assays, *A. stephensi* embryonic (ASE) cells were transfected with plasmids with NF-κB-dependent promoters from Defensin or Gambicin genes fused to a luciferase reporter gene as described [14], then stimulated with lipopolysaccharide (LPS) with or without 0.013 or 0.13 µM IGF1 and assayed for luciferase activity. Neither concentration of IGF1 affected promoter-reporter activity relative to treatment with LPS alone (Figure 2A, Figure S1), suggesting that IGF1 does not induce NF-κB-dependent gene expression in *A. stephensi* cells. We next assessed the effects of IGF1 on the expression of imd-associated immune effector genes APL1, LRIM, TEP1, and LRRD7 [37] *in vivo* using quantitative RT-PCR (Figure 2B). We have shown previously that *A. stephensi* fed artificial blood meals supplemented with freeze-thaw *P. falciparum* parasite products (FTPP) have increased expression of immune genes in the midgut epithelium 24 h post-blood feeding (9). Therefore, we fed female *A. stephensi* blood meals supplemented with FTPP and 0.013 µM or 0.13 µM IGF1 or FTPP and an equivalent volume of PBS (diluent for IGF1) as a control. IGF1 in the physiologically low range (0.013 µM) did not alter expression of these immune genes at 24 h post-feeding and a higher physiological concentration of IGF1 (0.13 µM) markedly reduced *APL1* and *TEP1* transcript levels despite activation of JNK (Figure 1). Therefore, IGF1 did not induce NF-κB-dependent promoter activity in *A. stephensi* cells *in vitro* (Figure 2A, Figure S1) and showed a trend towards repressed expression of imd-associated complement-like immune factors in the midgut *in vivo* (Figure 2B).

### Human IGF1 increased midgut RNOS in *A. stephensi*

JNK activation has also been linked to regulation of heme peroxidase 2 and nicotinamide adenine dinucleotide phosphate (NADPH) oxidase 5, which have been reported to function with NOS to opsonize parasites through TEP1-mediated lysis in *A. gambiae* [43]. Additionally, repression of midgut ERK phosphorylation by low and high IGF1 [17] could also act to enhance midgut *NOS* expression [8]. Together, these observations suggested that IGF1-mediated JNK activation and ERK repression could coordinately fuel RNOS synthesis and NO-dependent parasite killing in *A. stephensi*. To determine whether human IGF1 increased midgut RNOS, we quantified soluble peroxides, mitochondrial superoxide, and *NOS* gene expression levels in dissected midgut tissue following treatment with low (0.013 µM) and high (0.13 µM) IGF1.

**Figure 3:**
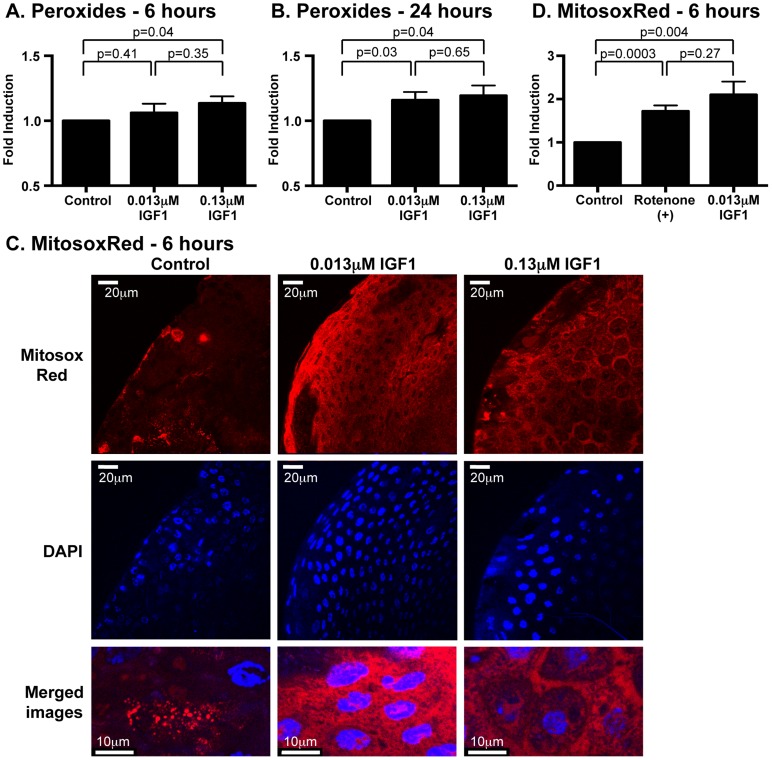
Ingested human IGF1 increased ROS levels in *A. stephensi* midguts. Peroxide levels were quantified at 6(A) and 24 h (B) following IGF1 treatment. Each data point represents the quantified peroxides in a pool of five midguts; three pools were collected per treatment per experiment. Data are represented as fold-change relative to saline/ATP-fed controls and were analyzed by unpaired t-test. Experiments were replicated three times with separate cohorts of mosquitoes and p-values are indicated. (C) Representative images from confocal microscopy of MitoSOX Red stained midguts. Upper Panel: MitoSOX Red staining, scale bars = 20 µm; Middle Panel: DAPI staining scale bars = 20 µm; Lower Panel: Merged images, scale bars = 10 µm. Differences in nuclei size are due to differences in focal plane not magnification. (D) Mitochondrial superoxide levels were quantified from MitoSOX Red fluorescence. Average fold inductions±SEM of MitoSOX Red fluorescence from low IGF1 (0.013 µM) and a positive control (1 µM rotenone) relative to controls. Data were analyzed by unpaired t-test (n = 4).

At 6 h post-treatment, high IGF1 significantly increased midgut peroxides, and by 24 h, both low and high IGF1 treatments increased midgut peroxide levels relative to controls (Figures 3A, B). Superoxide generated by mitochondria as a consequence of oxidative phosphorylation is the major cellular source of these ROS and superoxide dismutase converts mitochondrial superoxide to hydrogen peroxide [44], which can freely diffuse out of mitochondria. To determine whether the observed increases in midgut soluble peroxides originated from increased mitochondrial superoxide, we quantified superoxide levels in the mitochondria using MitoSOX Red, a selective fluorogenic dye that reacts specifically with mitochondrial superoxide [45]. At 6 h following IGF1 treatment, confocal microscopy of stained midguts revealed punctate MitoSOX Red staining that was consistent with mitochondrial superoxide localization (Figure 3C; merged images). This staining was significantly increased above control by treatment with 0.013 µM IGF1 to levels that were comparable to treatment with rotenone, an inducer of mitochondrial superoxide production [44] (Figure 3D). Taken together, IGF1 increased midgut mitochondrial superoxide and peroxides within 24 h of treatment, a possible consequence of IGF1-induced mitochondrial energy biosynthesis [46].

**Figure 4:**
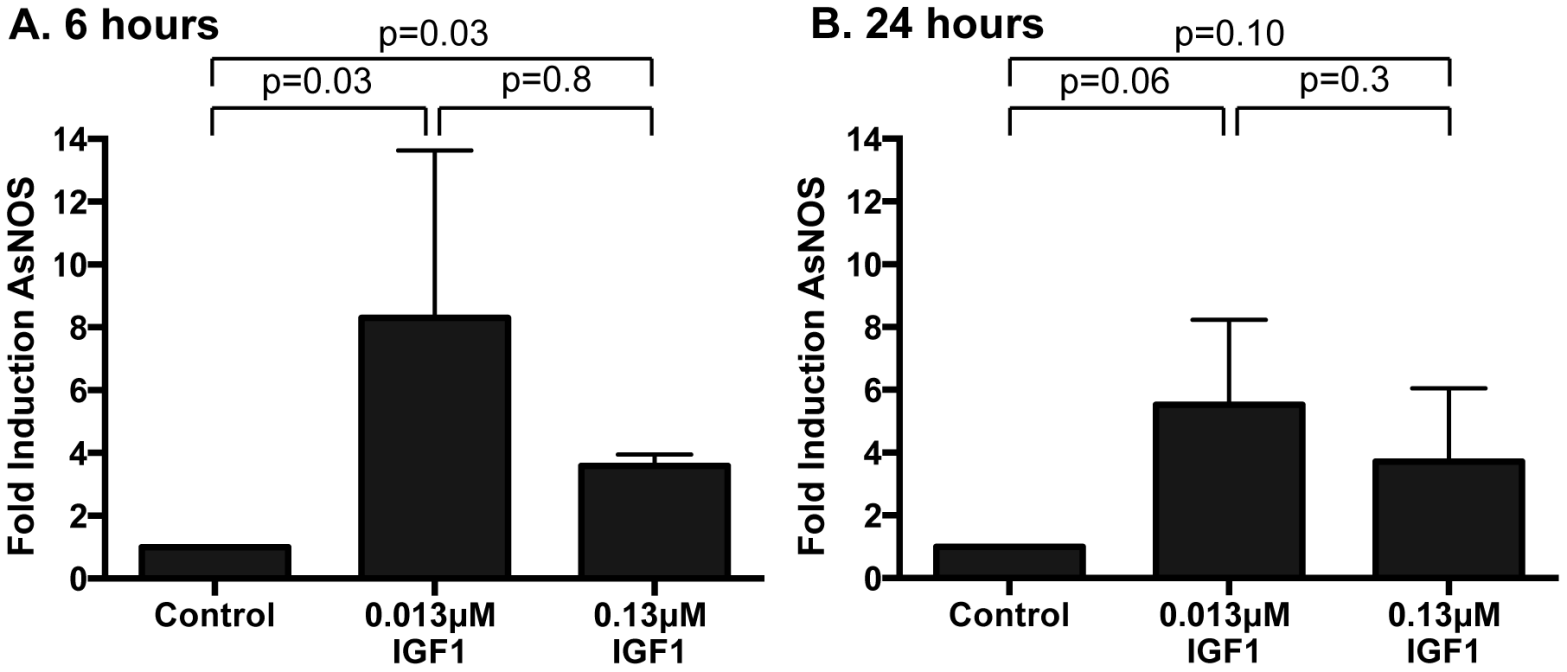
Ingested human IGF1 increased *NOS* gene expression in *A. stephensi* midguts. Mosquitoes were fed artificial blood meals supplemented with 0.013 or 0.13 µM IGF1 or with an equivalent volume of PBS as a control. Midguts were dissected at (A) 6 h and (B) 24 h after feeding and RNA was isolated for quantitative RT-PCR. Data are represented as fold change in *NOS* gene expression relative to PBS control. Experiments were replicated 4–6 times with separate cohorts of mosquitoes and p-values are indicated. Normally distributed data were analyzed by unpaired t-test (24 h), and non-normally distributed (6 h) data were analyzed by Mann Whitney test.

To determine whether human IGF1 concurrently increased midgut NOS levels, gene expression was quantified in midguts from *A. stephensi* females fed on artificial blood meals supplemented with low or high IGF1 (0.013 or 0.13 µM) or supplemented with an equivalent volume of PBS as a control at 6 h and 24 h following blood feeding. *NOS* expression was significantly higher in midguts of mosquitoes fed low and high IGF1 at 6 h post-blood meal (PBM) compared with blood-fed controls (Figure 4A). At 24 h PBM, *NOS* gene expression remained elevated in low and high IGF1-fed mosquitoes relative to controls, but variability in expression was greater at this timepoint (Figure 4B). Together with elevated levels of ROS (Figure 3), these data suggested that IGF1 stimulates the production of RNOS, which could underlie IGF1-mediated inhibition of parasite development [17].

### NOS inhibition reversed IGF1-induced resistance to *P. falciparum*

**Figure 5:**
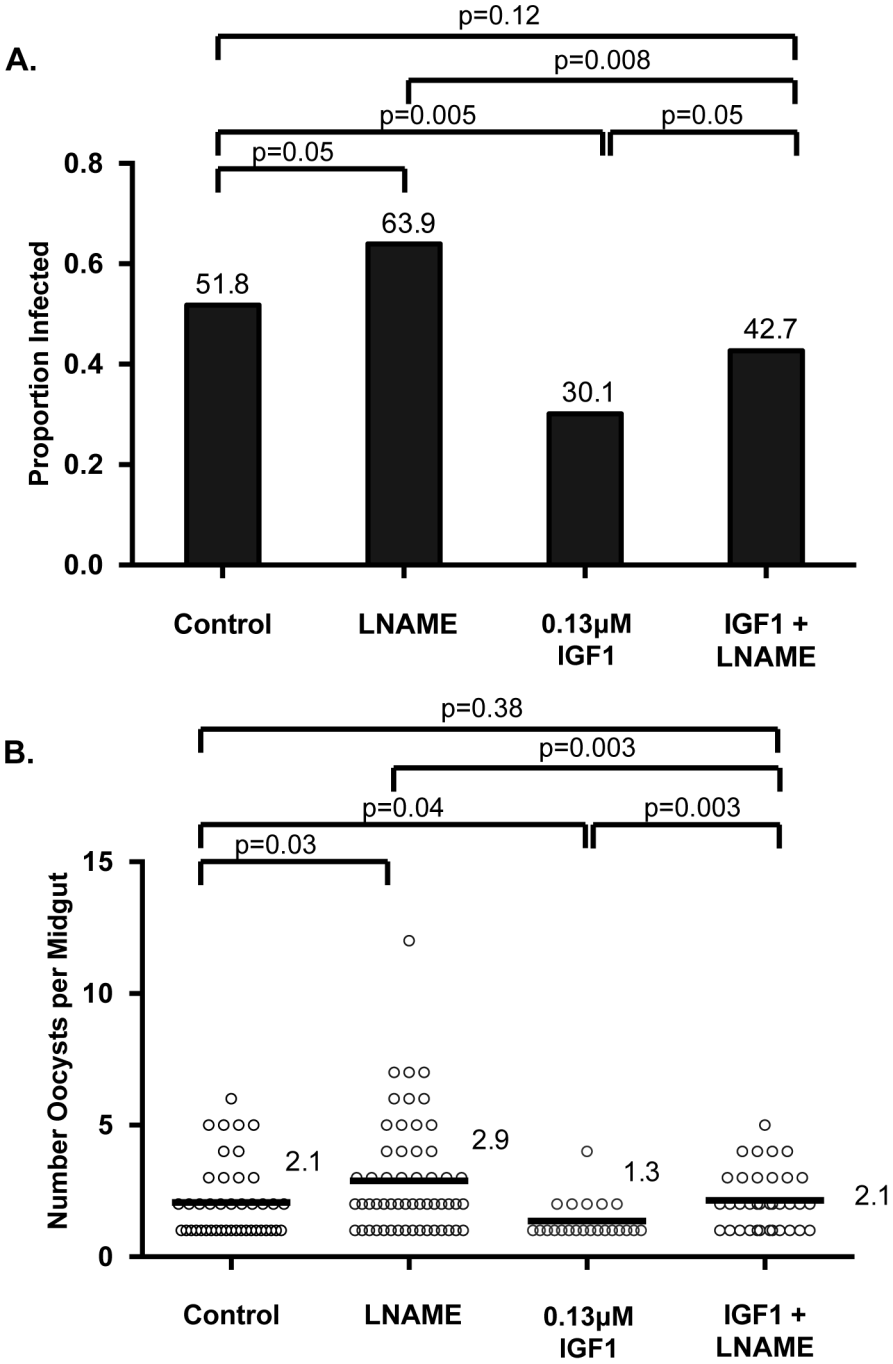
IGF1-induced resistance to *P. falciparum* was reversed by NOS inhibition. Age- and cohort-matched mosquitoes were fed infected blood meals supplemented with water, L-NAME, 0.13 µM IGF1 or 0.13 µM IGF1+L-NAME as described in Materials and Methods. This experiment was replicated four times with separate cohorts of mosquitoes. No significant differences in oocyst intensity were identified among replicate control groups (Kruskal-Wallis, p>0.05), so data were pooled for analysis. Intensity data shown are from infected midguts only. (A) Prevalence of infection (proportion of mosquitoes dissected with at least one *P. falciparum* oocyst; values indicated above bars). Fisher's exact test was used to compare treatments against L-NAME. (B) Intensity of infection (mean oocysts per infected midgut; values indicated to the right of data for each group). Mann-Whitney test was used to compare treatment groups to matched controls.

To assess whether RNOS were responsible for IGF1-induced resistance to *P. falciparum*, four separate cohorts of female *A. stephensi* were provided with water alone or water supplemented with 3.7 µM N**ω**-Nitro-L-arginine methyl ester (L-NAME), an inhibitor of NOS catalysis, for 72 h preceding infection. At the time of *P. falciparum* infection, mosquitoes were provided identical parasite-infected meals supplemented with or without 0.13 µM IGF1 and with or without 3.7 µM L-NAME. After feeding, infected mosquitoes were maintained on water or water with L-NAME as appropriate until dissection. At 10 days post-infection, L-NAME treatment in the absence of IGF1 increased infection prevalence (proportion of mosquitoes infected with at least one *P. falciparum* oocyst; Figure 5A) and intensity (mean oocysts per midgut; Figure 5B) relative to controls, consistent with previous observations [47]. As observed previously [17], treatment with IGF1 significantly reduced both prevalence and intensity of *P. falciparum* infection relative to control (Figures 5A, B). The addition of L-NAME to IGF1 treatment, however, reversed the phenotype of IGF1-associated infection resistance (IGF1+L-NAME vs IGF1: prevalence p = 0.05, intensity p = 0.003), resulting in prevalence of infection (Figure 5A) and mean oocysts per midgut (Figure 5B) in L-NAME+IGF1 treated mosquitoes that were not significantly different from control (IGF1+L-NAME vs control: prevalence p = 0.12, intensity p = 0.38).

### IGF1-induced RNOS are associated with enhanced caspase-3 activity and functional changes to the midgut epithelial barrier

**Figure 6:**
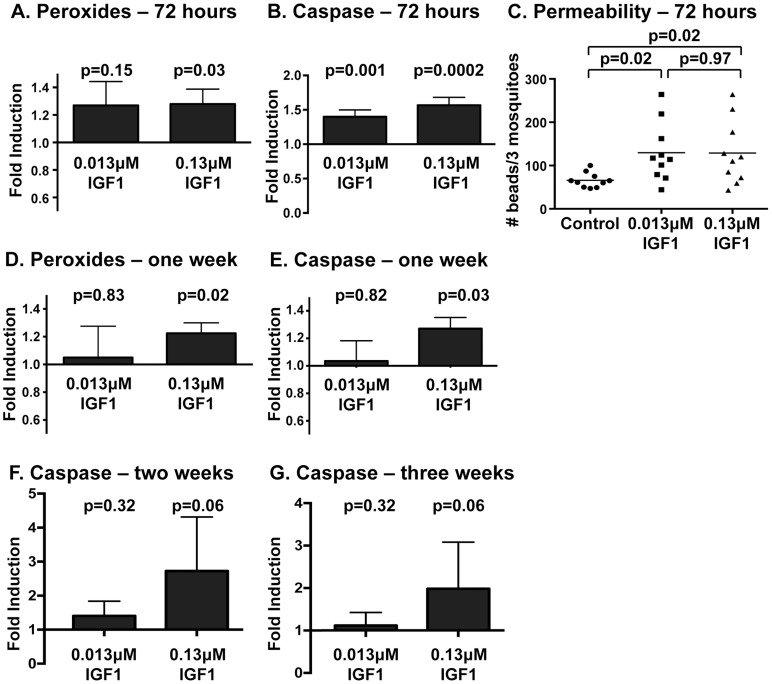
Midgut peroxides and epithelial integrity were altered at 72-IGF1 feeding. Peroxide levels in midguts at 72(A) and one week (D) following IGF1 treatment. Peroxides were quantified in pools of five midguts; three pools were collected per treatment per experiment. Experiments were replicated three times with separate cohorts of mosquitoes. Graphs show the mean fold induction of peroxides±SEM in IGF1 treated groups relative to saline/ATP-fed controls. Statistical significance was determined by unpaired t-test and p-values are indicated. Midgut caspase-3 activity at 72 h (B), one week (E), two weeks (F), and three weeks (G) following IGF1 treatment. Graphs show the average fold induction±SEM of AFC fluorescence (caspase-3 activity) relative to controls. Three pools of five midguts per treatment were collected for 72 h and one week timepoints and 1–2 pools were collected for two and three week timepoints. Experiments were replicated 4–6 times with separate cohorts of mosquitoes. Caspase-3 activity among groups was compared using Kolmogorov-Smirnov test. (C) Midgut permeability at 72 h following a blood-meal containing IGF1 and 3–3.5 µm fluorescent beads (Spherotech). Each data point represents the number of fluorescent beads in three whole mosquitoes quantified by flow-cytometry. Statistical significance was determined by one-way ANOVA followed by Fisher's LSD.

Our results indicate that IGF1-induced RNOS, rather than enhanced NF-κB-dependent innate immune responses, kill malaria parasites in the midgut of *A. stephensi*. While this may be beneficial for control of infection, sustained high levels of NO and ROS can also directly damage the gut epithelium and destabilize cellular junctions, which are critical to epithelial barrier function [31]. Based on these observations and our previous findings that IGF1 can extend lifespan in *A. stephensi* [17], we sought to assess midgut peroxides and epithelial integrity at extended times post-feeding of low (0.013 µM) and high (0.13 µM) IGF1. We observed that peroxides were significantly elevated by both treatments relative to control at 24 h (Figure 3B); at 72 h, only high IGF1 induced significant induction of peroxides relative to control (Figure 6A, Figure S2A). In addition, we assayed caspase-3 activity, a critical determinant of apoptotic regulation of epithelial barrier integrity [48]–[50], in midgut samples at 72 h. Caspase-3 activity was enhanced in both IGF1 treated groups compared to controls at 72 h (Figure 6B, Figure S2B), likely a reflection of the trend toward elevated peroxides with low IGF1 treatment and significant induction of peroxides with high IGF1 treatment at this timepoint (Figure 6A, Figure S2A). Elevated caspase-3 activity in midguts from both treatment groups suggested the occurrence of damage that could alter the physical barrier integrity of the midgut. Indeed, when we measured barrier integrity as the transit of fluorescent beads through the midgut at 72 h post-feeding, body bead counts (whole mosquito beads minus midgut beads) from both IGF1 treatment groups were increased significantly relative to controls (Figure 6C). Hence, IGF1-induced midgut ROS synthesis through 72 h was associated with an upregulation of caspase-3 activity and tissue damage as evidence by increased midgut permeability.

Our previous studies reported that survivorship of 0.013 µM IGF1-fed mosquitoes was greater than that of 0.13 µM IGF1-fed mosquitoes and untreated controls beginning at two weeks post-treatment. This divergent pattern was sustained for the duration of mosquito lifespan [17], suggesting that the effects of IGF1 on lifespan derive from distinct temporal effects of IGF1 on midgut homeostasis. This inference was supported by our data. In particular, midgut peroxide levels at one week post-feeding (Figure 6D, Figure S2C) remained significantly higher relative to controls in 0.13 µM IGF1-fed mosquitoes, while levels in 0.013 µM IGF1-fed mosquitoes had returned to baseline. Further, by one week post-feeding, midgut caspase-3 activity closely mirrored midgut peroxide levels in both IGF1 treatment groups (Figure 6E, Figure S2D), suggesting a link between the transient ROS produced in response to physiologically low levels of IGF1 and mechanisms of damaged midgut repair. In contrast, caspase-3 activity was sustained up to 3 weeks post-treatment with high IGF1 (Figures 6F and G, Figures S2E and F), suggesting that a lack of enhanced survivorship in this treatment group [17] resulted from sustained midgut damage.

### IGF1 regulates autophagy and ISC homeostatic processes in a concentration dependent manner

Several studies have suggested that autophagic repair is necessary for optimal stem cell maintenance and differentiation [51], [52]. These processes, in turn, facilitate enhanced pathogen resistance [25], [53], [54] and epithelial barrier integrity and repair functions in the gut [55], [56]. To determine whether the IGF1 phenotypes we observed were associated with expression patterns typical of ISC proliferation and differentiation we examined transcript levels for *Esg* (proliferation) and *Pros* (differentiation) over time in the midgut epithelium of *A. stephensi* [31], [57], [58] fed artificial blood meals supplemented with low and high IGF1 or supplemented with an equivalent volume of PBS as a control. To monitor the formation and maturation of autophagosomes, we also examined transcript levels of *ATG6* (autophagosome initiation; [59]) and *ATG8* (maturation of the autophagosomal membrane; [59]) as previously described [31].

*Esg* expression was significantly decreased relative to control at 24 h (p = 0.0003), but levels remained relatively unchanged from control at 72 h and one week post-treatment (Table 1). From 24 h through one week post-treatment with low IGF1, *Pros* expression levels were relatively unchanged from control (Table 1). In contrast, treatment with high IGF1 was associated with significant increases of 11-fold for *Esg* (p = 0.0003) by 72 h and 74-fold for *Pros* by one week (p = 0.002) relative to control (Table 1). These data suggested that enteroblast proliferation and differentiation are maintained at steady-state levels by low IGF1 treatment, while high IGF1 treatment was associated with a marked rise in proliferation and differentiation starting at 72 h PBM. Autophagy biomarker gene expression patterns were also distinct between these two IGF1 treatments. In particular, low IGF1 treatment induced moderate increases (2-fold relative to control) in *ATG6* expression at one week post-treatment (Table 1; p = 0.06) and a greater than 19-fold increase in *ATG8* expression relative to control at 24 h (p = 0.02) that was not sustained at 72 h and one week post-treatment (Table 1). Treatment with high IGF1 moderately increased *ATG6* expression at 72 h post-treatment (p = 0.06, Table 1). However, *ATG8* expression levels with high IGF1 treatment differed from that with low IGF1 in that a smaller significant induction (4.65-fold, p = 0.03) relative to control was noted at 72 h (Table 1), and by one week post-treatment, *ATG8* expression levels were only 29% of control levels (p = 1.62E-07, Table 1). These data suggested that maturation of autophagosomes was enhanced early and significantly by low IGF1, whereas this process was transiently enhanced at 72 h and then significantly repressed by high IGF1 by one week post-treatment.

**Table 1:** IGF1 regulates autophagy and ISC homeostatic processes in a concentration dependent manner.

	0.013 µM IGF1			0.13 µM IGF1			
	24 h	72 h	1 wk	24 h	72 h	1 wk	
**Esg**	**0.32**	2.62	0.61	0.20	**11.12**	2.11	**geometric mean**
	**0.0003**	0.44	0.25	0.35	**0.0003**	0.20	**p-value**
**Pros**	1.31	7.88	2.22	1.92	2.00	**74.39**	**geometric mean**
	0.19	0.17	0.87	1.00	0.99	**0.002**	**p-value**
**ATG6**	0.76	3.36	**2.06**	2.58	**2.22**	3.83	**geometric mean**
	0.49	0.33	**0.06**	0.23	**0.06**	0.15	**p-value**
**ATG8**	**19.75**	6.53	0.11	14.01	**4.65**	**0.29**	**geometric mean**
	**0.02**	0.99	1.00	0.26	**0.03**	**1.62E-07**	**p-value**

Gene expression levels were calculated using the 2^−ΔΔCt^ method relative to expression of the ribosomal protein s7 (RPS7) gene. Values indicate geometric mean fold changes between IGF1-fed and buffer-fed controls. Bold values indicate significant (p<0.05) or nearly significant (p = 0.06) fold changes in expression relative to control.

### The beneficial effects of low dose IGF1 on *A. stephensi* survivorship was ablated by enhanced oxidative stress

**Figure 7:**
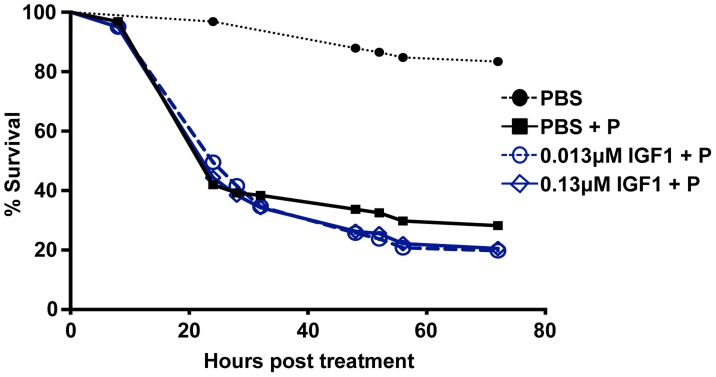
Effect of low dose IGF1 on *A. stephensi* survivorship was ablated by enhanced oxidative stress. Mosquitoes were fed saline/ATP supplemented with: (1) low (0.13 µM) IGF1 or high (0.13 µM) IGF1 and 1 mM paraquat (P), (2) an equivalent volume of PBS and 1 mM paraquat (PBS+P), or (3) an equivalent volume of PBS without paraquat (PBS−P) as an untreated control. Dead mosquitoes were removed and counted at intervals between 8 h and 72 h. The experiment was replicated twice with separate cohorts of mosquitoes. Survival analyses were performed using the Kaplan Meier method and differences between survival curves calculated using the Wilcoxon test. Survival curves for 0.13 µM IGF1+P and 0.13 µM IGF1+P were not significantly different from each other and neither was significantly different from PBS+P (p>0.05). Survival curves for low IGF1+P, high IGF1+P, and PBS+P, however, were significantly different from the untreated control PBS−P (p<0.05).

Our data suggest that low IGF1 treatment induces transient oxidative stress and caspase-3 activity (Figure 6) as well as early enhanced autophagy and maintenance of homeostatic levels of epithelial proliferation and differentiation (Table 1) that are consistent with lifespan extension [17]. Conversely, high IGF1 treatment induced prolonged oxidative stress (Figure 6), sustained midgut caspase-3 activity (Figure 6), and aberrant expression patterns of epithelial cell proliferation, differentiation, and autophagy biomarkers (Table 1). These data suggested that the lack of lifespan extension observed in high IGF1 treated mosquitoes was due a reduced capacity to recover from oxidative stress. To address this hypothesis, 3–5 day old female *A. stephensi* were provided reconstituted human blood meals supplemented with 1 mM paraquat, an inducer of mitochondrial ROS [44], 1 mM paraquat and low or high IGF1, or supplemented with diluent at volumes equivalent to those of the treatment groups. Because paraquat acutely induces oxidative stress, our observations were temporally restricted to 72 h post-treatment, a time at which midgut peroxides following low IGF1 treatment were no longer significantly elevated relative to controls (Figure 6). We expected that capacity for recovery from oxidative stress within the period of observation, therefore, would be greater in the low IGF1 treatment group. However, the addition of low and high IGF1 to paraquat treatment resulted in nearly identical reductions in survivorship relative to paraquat alone (Figure 7). These observations suggested that the beneficial effects of low IGF1 on lifespan extension of *A. stephensi* were not due to a greater capacity for recovery from oxidative stress, which would mitigate paraquat-induced stress, but rather to the lower levels of midgut epithelial ROS induced by low versus high IGF1.

## Discussion

Previously, we showed that physiological concentrations of human IGF1 reduced *P. falciparum* development in *A. stephensi*, and that low IGF1 (0.013 µM) extended mosquito lifespan while high IGF1 (0.13 µM) did not [17]. By extension, mechanisms of parasite killing should be shared by low and high IGF1 concentrations, while lifespan extending effects of IGF1 should be unique to low concentrations of this growth factor. We focused our studies accordingly and assert that our observations not only explain these divergent effects of IGF1, but also extend our understanding of the intersection of lifespan and pathogen resistance as mediated by midgut IIS.

Here we have demonstrated that both low and high IGF1 induced midgut *NOS* expression at 6 h and 24 h post-feeding (Figure 4), although low IGF1 may have done this more effectively, via enhanced JNK activation, than high IGF1 treatment (Figure 1). Regardless, inhibition of NOS activity by L-NAME reversed IGF1 inhibition of *P. falciparum* development (Figure 5). Parasite killing, therefore, is mediated through toxic RNOS [7] formed in the presence of IGF1-induced NO and ROS in the midgut lumen. Hence, although JNK activation may mediate anti-parasite defenses beyond those examined here, their contribution is clearly masked by a dominant NO-mediated defense.

While the mechanism of IGF1-mediated control of parasite infection was reasonably clear, our data suggested that the divergent effects of low and high IGF1 on *A. stephensi* survivorship and lifespan were more complex. Key observations included enhanced JNK activation that was specific to low IGF1 (Figure 1), significant inductions in mitochondrial ROS and peroxides produced in the midgut that were sustained only with high IGF1 (Figures 3, 6), and aberrant expression patterns of epithelial cell proliferation, differentiation, and autophagy biomarkers with high IGF1 treatment (Table 1). We also observed high IGF1-induced midgut caspase-3 activity for up to 3 weeks post-treatment (Figure 6), a time during which differences in survivorship of *A. stephensi* treated with low and high IGF1 were significant [17].

**Figure 8:**
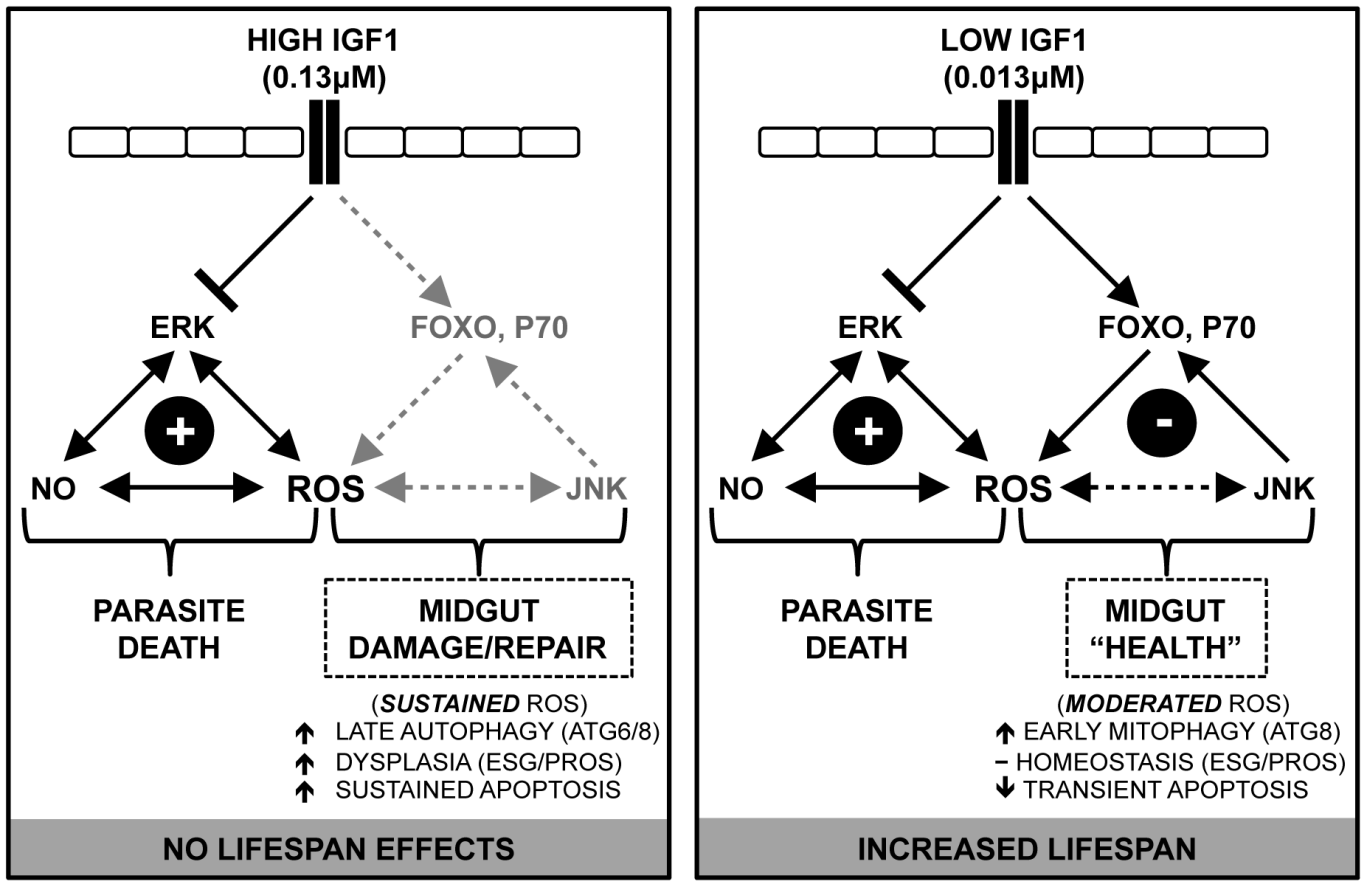
Proposed model: IGF1 fine-tunes the balance of epithelial homeostasis and midgut integrity, enhancing survival and anti-parasite resistance in *A. stephensi*. Human IGF1 in the midgut initiates signaling cascades that repress phosphorylation of ERK, enhancing midgut RNOS that induce parasite death. Low IGF1 acts through FOXO, JNK and P70S6K to induce JNK/IIS-mediated cytoprotective responses that moderate levels of ROS and NO to initiate early mitophagy, transient apoptosis, and ISC homeostasis, which increase mosquito survivorship. In contrast, high IGF1 does not activate JNK/IIS compensatory signaling, resulting in delayed autophagy and sustained RNOS, which drive damage-associated apoptosis and midgut epithelial dysplasia.

Importantly, these phenotypes correspond to the dichotomy in IGF1-stimulated phosphorylation of IIS proteins, namely, that only low IGF1 induced phosphorylation of FOXO and p70S6K in *A. stephensi* midguts [17]. We propose that FOXO and p70S6K activation induce cytoprotective responses that ameliorate damage and protect and expand mitochondrial function, resulting in increased mosquito lifespan. In particular, activation of p70S6K is critical to inhibiting BCL2-mediated apoptosis [21], which could explain the attenuation of midgut caspase-3 activity observed in *A. stephensi* following low dose IGF1 treatment. Further, only low IGF1 significantly activated JNK, which in turn could enhance pleiotropic cytoprotection beyond JNK-mediated midgut host defense, including targeting of Akt-phosphorylated FOXO to activate an antioxidant program that reduces cellular ROS [60], [61]. JNK-mediated cytoprotection would also be consistent with homeostatic ISC biomarker expression in the *A. stephensi* midgut following low IGF1 treatment. In *Drosophila*, ISCs are mostly quiescent and become highly proliferative following injury [30], [62]. In contrast, midgut epithelium from *A. stephensi* treated with low IGF1 showed a small but significant change in *Esg* expression, suggesting that midgut cells experience little, if any, injury from enhanced RNOS. Over the lifetime of *A. stephensi*, patterns of transient ROS induction coupled with early epithelial recovery could facilitate midgut homeostasis that would be consistent with enhanced survivorship in the context of parasite killing (Figure 8).

The possibility that transient levels of ROS are beneficial to *A. stephensi* is supported by recent observations in *D. melanogaster*. In particular, Owusu-Ansah et al. [63] showed that moderate levels of oxidative stress in muscle mitochondria induced mitohormesis or adaptive cytoprotective responses that preserve mitochondrial function and extend lifespan in *D. melanogaster*. Importantly, these protective effects were due to the actions of (i) JNK, which induced the mitochondrial unfolded protein response (UPR) preserving mitochondrial function and (ii) induction of ImpL2 (the *Drosophila* homologue of IGF-binding protein 7), which enhanced muscle mitophagy and repressed systemic insulin signaling, preserving tissue integrity and contributing to extended lifespan. These observations suggest that JNK-mediated feedback to IIS could reduce mitochondrial ROS to “optimal” levels in the mosquito midgut in response to low IGF1 treatment, which would promote UPR and preserve mitochondrial integrity. Early induction of ATG8 would also facilitate timely removal of moderately dysfunctional mitochondria, thus protecting the pool of optimally functioning organelles. Finally, while insulin-like peptide (ILP) binding proteins have yet to be identified in *A. stephensi*, proteins that can bind to mosquito ILP3 and 4 have been identified in *Aedes aegypti* [64], suggesting that ImpL2-like function could contribute to IGF1-mediated, IIS-dependent effects on lifespan and immunity in *A. stephensi*.

Generating genetically modified fit mosquitoes that resist malaria parasite infection is a central goal of malaria transmission control research. Our efforts to genetically manipulate IIS to regulate mosquito immunity and fitness have resulted in both short- and long-lived mosquitoes that can resist *P. falciparum* infection, phenotypes that are defined by patterns of epithelial homeostasis and midgut integrity. The results detailed here provide evidence that IGF1 can fine-tune the balance of these processes in the mosquito midgut, effecting enhanced survival and enhanced anti-parasite resistance. Further, our studies provide critical insights into manipulation of conserved IIS regulation of mitochondrial integrity and epithelial homeostasis for concurrent optimization of fitness and anti-pathogen resistance that can be broadly applied to mosquito-focused control strategies for malaria and other vector-borne diseases.

## Materials and Methods

### Mosquito cell culture, mosquito rearing, and experimental treatments

Immortalized, ASE cell lines were maintained as previously described [9]. For *in vivo* studies, *A. stephensi* Liston (Indian wild-type strain) were reared and maintained at 27°C and 75% humidity. All mosquito rearing and feeding protocols were approved and in accordance with regulatory guidelines and standards set by the Institutional Animal Care and Use Committee of the University of California, Davis. For most experimental treatments, laboratory-reared 3–5 day old female mosquitoes were maintained on water for 24–48 h and then allowed to feed for 30 min on reconstituted human blood meals (healthy male adult, blood type O+) provided through a Hemotek Insect Feeding System (Discovery Workshops, Accrington, UK). Reconstituted blood meals contained washed human red blood cells (RBCs) and saline (10 mM, NaHCO_3_, 15 mM NaCl, pH 7.0) as 50% RBCs and 50% saline with or without recombinant human IGF1. Human RBCs were purchased from Interstate Blood Bank (Memphis, TN, USA). Recombinant human IGF1 was purchased from R&D Systems (Minneapolis, MN, USA).

### Western blotting

Female 3–5 day old mosquitoes were maintained on water for 24 h and then allowed to feed for 30 min on a reconstituted blood meal supplemented with low (0.013 µM) IGF1, high (0.13 µM) IGF1 or with phosphate-buffered saline (PBS) as a control. Protocols for analysis of protein phosphorylation have been described previously [17]. In brief, protein lysates from dissected midguts from 30 blood fed mosquitoes per treatment group were probed using primary and secondary antibodies at the following dilutions: 1∶1,250 phospho-JNK (1°; Biosource, Carlsbad, CA)/1∶20,000 rabbit anti-mouse IgG (2°; Sigma-Aldrich, St. Louis, MO) and 1∶10,000 GAPDH (1°; Abcam, San Francisco, CA)/1∶20,000 goat anti-rabbit IgG (2°; Cell Signaling Technology, Danvers, MA). Blots were developed using SuperSignal West Dura chemiluminescent reagent (Thermo Fisher Scientific, Rockford, IL), and visualized using the Kodak Image Station 4000MM Pro and Molecular Imaging software (Carestream Health, Rochester, NY). Experiments were replicated four times with separate cohorts of mosquitoes. Data were analyzed by Mann Whitney test (Graphpad Prism 6.0).

### Cell culture, transfection, and luciferase reporter assays

Defensin and Gambicin promoters were cloned into the promoter-less luciferase reporter pGL3 (Promega; Madison, Wisconsin). Transfections of *A. stephensi* ASE cells were performed using Effectene transfection reagent (Qiagen; Valencia, CA) as previously described [14]. At 24 h post-transfection, cells were challenged with 100 µg/ml LPS (*Escherichia coli* serotype 026:B6; Sigma-Aldrich) with or without 0.013–1.3 µM recombinant human IGF1 (Sigma-Aldrich). Luciferase activity was measured by microplate reader (Hidex Chameleon, LabLogic; Brandon, FL) using the Dual-Glo Luciferase Assay (Promega) at 24 h post-immune challenge. Data were analyzed by unpaired t-tests comparing each treatment to matched buffer controls. Experiments were replicated three (Gambicin) or four (Defensin) times.

### Preparation of *P. falciparum* freeze-thaw parasite product (FTPP)

*P. falciparum* FTPP was used to induce uniform signaling events in *A. stephensi* cells *in vivo*. Preparation and feeding of FTPP to *A. stephensi* were described in detail in [9], [32]. In brief, FTPP was prepared from 15 day old cultures (or when stage V gametocytes were evident) of *P. falciparum*-infected RBCs subjected to three freeze/thaw cycles of −80°C for 10 min followed by 37°C for 10 min. To mimic parasite infection, FTPP was diluted with uninfected human RBCs and heat-inactivated human serum (type A+, Interstate Blood Bank). As a control, an equal volume of uninfected RBCs was similarly frozen and thawed (FT-RBC) and diluted with intact uninfected RBCs and heat-inactivated human serum (type A+). All blood meals were 50% FTPP/RBCs and 50% human serum. Female mosquitoes were fed with FTPP and FT-RBC supplemented with low (0.013 µM) and high (0.13 µM) IGF1 or with PBS as a control, and processed for immune gene expression analysis by qRT-PCR as described below. Experiments were replicated six times with separate cohorts of mosquitoes. Data were analyzed using Kruskal-Wallis followed by Dunn's post-test.

### Quantitative reverse-transcriptase-PCR analyses (qRT-PCR)

Female mosquitoes (3–5 d old) were maintained on water for 24 h and then allowed to feed for 30 min on a reconstituted blood meal supplemented with low (0.013 µM) IGF1, high (0.13 µM) IGF1 or PBS as a control, or FTPP/FT-RBC similarly supplemented as described above. At various times post-blood feeding, a total of 25–30 midguts were dissected into RNA-later (Life Technologies, Grand Island, NY) from each treatment group per experiment. Dissected midguts were homogenized in TRIZOL (Life Technologies) and RNA was extracted according to the manufacturer's protocol. cDNAs were synthesized from RNA samples using the SuperScript III First-Strand Synthesis System (Life Technologies). Quantitative RT-PCR was performed using Maxima SYBR green/ROX qPCR Master Mix (Thermo Fisher Scientific) on an ABI 7300 Sequence Detection System (Life Technologies). Expression levels were calculated using the 2^−ΔΔCt^ method relative to the ribosomal protein s7 (RPS7) gene. Relative transcript levels for *RPS7*, *LRIM*, *LRRD7*, *TEP1*, *APL1*, and *NOS* were determined using primers and cycling conditions described in [32]. For qRT-PCR assays for transcript levels for *ATG6*, *ATG8*, *Pros*, and *Esg*, cDNA samples were pre-amplified using gene specific primers prior to qPCR as described in [31]. Experiments were replicated 4–6 times with separate cohorts of mosquitoes. Normally distributed data were analyzed by unpaired t-test, and non-normally distributed data were analyzed by Mann Whitney test relative to matched controls.

### ROS assays

For detection of superoxide, groups of 50 (3–5 d old) female *A. stephensi* mosquitoes were held on water overnight, then allowed to feed for 30 min on an ATP-saline solution (150 mM NaCl, 10 mM NaHCO_3_ and 1 mM ATP) supplemented with equivalent volumes of one of the following: PBS as a control, rotenone (1 µM) to induce the formation of superoxide as a positive control, or human IGF1. Non-fed mosquitoes were removed at the end of 30 min. At 6 h post feeding, two pools of 10 midguts for each treatment were dissected and incubated in 150 µl of 5 µM MitoSOX Red (Life Technologies) in wells of a 96-well plate. The plate was incubated in the dark at room temperature for 30 min and then read at excitation 485 nm and emission 560 nm (Hidex Chameleon, LabLogic). Non-fed mosquito midguts were used to define baseline superoxide levels. Confocal images were prepared from additional midguts from the same feeding groups. For microscopy, midguts were mounted in Prolong Gold with DAPI (Life Technologies), imaged using an Olympus FV1000 confocal microscope (Olympus; Center Valley, PA) and processed using ImageJ [65] and Adobe Photoshop. Treatment data were analyzed by unpaired t-test relative to matched controls. Experiments were replicated four times with separate cohorts of mosquitoes. For detection of midgut peroxides, pools of 5 dissected midguts in 50 µL PBS (two per treatment) were homogenized by brief sonication and centrifuged to pellet debris. Peroxides were measured in 20 µL of sample supernatant in duplicate using the Pierce Quantitative Peroxide assay kit (Thermo Fisher Scientific) as per manufacturer's instructions. Samples were incubated for 20 min at room temperature prior to spectrophotometer reading. To quantify peroxide levels at 6 h and 24 h, when blood in the midgut would confound the assay, mosquitoes were fed on a ATP-saline solution (150 mM NaCl, 10 mM NaHCO_3_ and 1 mM ATP) containing the following treatments: PBS control, low IGF1 (0.013 µM) or high IGF1 (0.13 µM). For each assay, three pools of five midguts per treatment were collected and processed as described above. Peroxide assays were replicated three times (blood-fed and saline-fed) with separate cohorts of mosquitoes and treatment data were analyzed by unpaired t-test relative to matched controls.

### Caspase-3 activity assay

Female 3–5 day old *A. stephensi* were fed reconstituted human blood meals supplemented with equivalent volumes of PBS, low IGF1 (0.013 µM) or high IGF1 (0.13 µM) and midgut caspase-3 activity was measured at various times following treatment. For extended times (two and three weeks), mosquitoes were fed once per week on supplemented blood meals (PBS or IGF1) and allowed to oviposit once per week. Pools of 5 midguts per treatment were placed in 50 µL PBS; 3 pools were collected for 72 h and one week timepoints and 1–2 pools were collected for two and three week timepoints. Midguts were dissected, homogenized by brief sonication, and centrifuged to pellet debris. A total of 25 µL of supernatant was added to 25 µL PBS with 1 mM DTT, and 10 µL of the resulting solution was analyzed in duplicate using the caspase-3 fluorometric substrate DEVD-AFC (Biovision, Milpitas, CA) per manufacturer's instructions. An additional 10 µL of sample was treated with the caspase-3 inhibitor Z-DEVD-FMK (Biovision) for a negative control. Samples were incubated at 37°C in the dark and fluorescence readings were taken at 30 min and 2 h after reagents were added. Experiments were replicated 4–6 times with separate cohorts of mosquitoes. Caspase-3 activity among groups was compared using Kolmogorov-Smirnov test.

### Functional assay of midgut permeability in IGF1-fed *A. stephensi*

Female 3–5 day old *A. stephensi* were kept on water for 48 h and then allowed to feed for 30 min on reconstituted human blood meals supplemented with equivalent volumes of low (0.013 µM) IGF1, high (0.13 µM) IGF1 or PBS as a control together with 1×10^6^ fluorescent beads/ml (3.0–3.4 µm, Sphero Rainbow Calibration particles RCP-30-5A-2; Spherotech, Lake Forest, IL) as described [31]. Non-fed mosquitoes were removed immediately after feeding. At 72 h post-feeding, samples of three whole mosquitoes or three dissected midguts were placed in PBS, pulse sonicated, and filtered through a 35 µm nylon mesh to remove tissue debris. Sample data were acquired with a FACScan flow cytometer (BD Biosciences, San Jose, CA) and analyzed using FlowJo software (version 6.4.1; Tree Star). To remove the contribution of beads remaining in the midgut from the whole body bead count the beads in three midguts (pooled) were quantified and subtracted from each analyzed sample of three whole mosquitoes (pooled). Five replicates of three whole pooled mosquitoes were analyzed per experiment. Experiments were replicated with two separate cohorts of mosquitoes. Statistical significance was determined by one-way ANOVA followed by Fisher's LSD.

### Malaria parasite infection/L-NAME treatment

*P. falciparum* NF54 strain was cultured in 10% heat-inactivated human serum and 6% washed human RBCs in RPMI 1640 with HEPES (Life Technologies) and hypoxanthine for 15 days or until stage V gametocytes were evident. Exflagellation rates of mature gametocytes were evaluated on the day prior to and the day of mosquito infection. Mosquitoes were fed on mature gametocyte cultures diluted with human RBCs and heat-inactivated human serum (final concentration 50% RBCs, 50% serum) for 30 min. Protocols for the culture and handling of *P. falciparum* for mosquito feeding were approved and in accordance with regulatory guidelines and standards set by the Biological Safety Administrative Advisory Committee of the University of California, Davis.

For NOS inhibition, 125 female *A. stephensi* were provided with water only, with 3.7 µM L-NAME (Sigma-Aldrich), or with 0.13 µM IGF1 plus L-NAME from 72 h before blood feeding, at the time of *P. falciparum* infection (supplemented into the blood meal), and thereafter until dissection. After 10 days, fully gravid females were dissected and midguts were stained with 0.1% mercurochrome to visualize *P. falciparum* oocysts. Oocysts were counted for each midgut and mean oocysts per midgut (infection intensity) and percentages of infected mosquitoes (infection prevalence; infection defined as at least one oocyst) were calculated for all dissected mosquitoes. This experiment was repeated four times with separate cohorts of mosquitoes. Infection intensity data were analyzed by Kruskal-Wallis test to determine whether the oocysts per midgut in the controls differed among replicates. Since no differences were evident, the data were pooled across replicates. Oocyst counts were compared using the Mann-Whitney test for nonparametric data. Infection prevalence data were analyzed by Fisher's exact test to determine whether infection status differed between treatment conditions.

### Oxidative stress survivorship assays

100 female 3–5 day old *A. stephensi* were kept on water for 48 h, and then fed for 30 min on a saline/ATP solution (150 mM NaCl, 10 mM NaHCO_3_, 1 mM ATP) supplemented with 1 mM paraquat (Sigma-Aldrich) and equivalent volumes of PBS, low IGF1 (0.013 µM) or high IGF1 (0.13 µM). Dead mosquitoes were removed and counted at various times between 8 h and 72 h. The experiment was replicated twice with separate cohorts of mosquitoes. Survival analyses were performed using the Kaplan Meier method and differences between survival curves calculated using the Wilcoxon test.

## Supporting Information

Figure S1 **Raw (non-normalized) data from** **Figure 2A** **.** Human IGF1 did not alter antimicrobial peptide (AMP) promoter activity in immune-activated ASE cells. Graphs depicts relative light units (RLU) from promoter-reporter assays in transfected cells stimulated with or without LPS and with or without IGF1.
(TIF)

Figure S2 **Raw (non-normalized) data from** **Figure 6** **.** Peroxide levels in midguts at 72 h (A) and one week (C) following IGF1 treatment. Peroxides were quantified in pools of five midguts; three pools were collected per treatment per experiment. Experiments were replicated three times with separate cohorts of mosquitoes. Graphs show the µM H_2_O_2_/midgut. Midgut caspase-3 activity at 72 h (B), one week (D), two weeks (E), and three weeks (F) following IGF1 treatment. Graphs show AFC fluorescence (caspase-3 activity). Three pools of five midguts per treatment were collected for 72 h and one week timepoints and 1–2 pools were collected for two and three week timepoints. Experiments were replicated 4–6 times with separate cohorts of mosquitoes.
(TIF)

## References

[1] World Health Organization (2012) World malaria report 2012. World Health Organization.

[2] Enayati A, Hemingway J (2010) Malaria management: past, present, and future. Ann Rev Entomol 55: 569–591. 19754246 10.1146/annurev-ento-112408-085423

[3] Whitten MM, Shiao SH, Levashina EA (2006) Mosquito midguts and malaria: cell biology, compartmentalization and immunology. Parasite Immunol 28: 121–130. 16542314 10.1111/j.1365-3024.2006.00804.x

[4] Clayton AM, Dong Y, Dimopoulos G (2013) The *Anopheles* innate immune system in the defense against malaria infection. J Innate Immun 6: 169–81 DOI: 10.1159/000353602 23988482 10.1159/000353602 PMC3939431

[5] Baton LA, Ranford-Cartwright LC (2012) Ookinete destruction within the mosquito midgut lumen explains *Anopheles albimanus* refractoriness to *Plasmodium falciparum* (3D7A) oocyst infection. Intl J Parasitol 42: 249–258. 10.1016/j.ijpara.2011.12.005 PMC3401372 22366731

[6] Luckhart S, Vodovotz Y, Cui L, Rosenberg R (1998) The mosquito *Anopheles stephensi* limits malaria parasite development with inducible synthesis of nitric oxide. Proc Natl Acad Sci U S A 95: 5700–5705. 9576947 10.1073/pnas.95.10.5700 PMC20442

[7] Peterson TM, Gow AJ, Luckhart S (2007) Nitric oxide metabolites induced in *Anopheles stephensi* control malaria parasite infection. Free Rad Biol Med 42: 132–142. 17157200 10.1016/j.freeradbiomed.2006.10.037 PMC1764505

[8] Surachetpong W, Singh N, Cheung KW, Luckhart S (2009) MAPK ERK signaling regulates the TGF-β1-dependent mosquito response to *Plasmodium falciparum* . PLoS Pathog 5: e1000366. 19343212 10.1371/journal.ppat.1000366 PMC2658807

[9] Pakpour N, Camp L, Smithers HM, Wang B, Tu Z, et al (2013) Protein kinase C-dependent signaling controls the midgut epithelial barrier to malaria parasite infection in anopheline mosquitoes. PLoS One 8: e76535. 24146884 10.1371/journal.pone.0076535 PMC3795702

[10] Lim J, Gowda DC, Krishnegowda G, Luckhart S (2005) Induction of nitric oxide synthase in *Anopheles stephensi* by *Plasmodium falciparum*: mechanism of signaling and the role of parasite glycosylphosphatidylinositols. Infect Immun 73: 2778–2789. 15845481 10.1128/IAI.73.5.2778-2789.2005 PMC1087374

[11] Pakpour N, Akman-Anderson L, Vodovotz Y, Luckhart S (2013) The effects of ingested mammalian blood factors on vector arthropod immunity and physiology. Microbes Infect 15: 243–254. 23370408 10.1016/j.micinf.2013.01.003 PMC3602389

[12] Kang MA, Mott TM, Tapley EC, Lewis EE, Luckhart S (2008) Insulin regulates aging and oxidative stress in *Anopheles stephensi* . J Exp Biol 211: 741–748. 18281336 10.1242/jeb.012955 PMC2592302

[13] Surachetpong W, Pakpour N, Cheung KW, Luckhart S (2011) Reactive oxygen species-dependent cell signaling regulates the mosquito immune response to *Plasmodium falciparum* . Antioxid Redox Signal 14: 943–955. 21126166 10.1089/ars.2010.3401 PMC3042311

[14] Pakpour N, Corby-Harris V, Green GP, Smithers HM, Cheung KW, et al (2012) Ingested human insulin inhibits the mosquito NF-κB-dependent immune response to *Plasmodium falciparum* . Infect Immun 80: 2141–2149. 22473605 10.1128/IAI.00024-12 PMC3370580

[15] White NJ, Warrell DA, Chanthavanich P, Looareesuwan S, Warrell MJ, et al (1983) Severe hypoglycemia and hyperinsulinemia in falciparum malaria. NE J Med 309: 61–66. 10.1056/NEJM198307143090201 6343877

[16] Planche T, Dzeing A, Ngou-Milama E, Kombila M, Stacpoole PW (2005) Metabolic complications of severe malaria. In Malaria: Drugs, Disease and Post-genomic Biology Berlin Heidelberg: Springer. pp. 105–136. 10.1007/3-540-29088-5_5 16265889

[17] Drexler A, Nuss A, Hauck E, Glennon E, Cheung K, et al (2013) Human IGF1 extends lifespan and enhances resistance to *Plasmodium falciparum* infection in the malaria vector *Anopheles stephensi* . J Exp Biol 216: 208–217. 23255191 10.1242/jeb.078873 PMC3597202

[18] Mizushima Y, Kato H, Ohmae H, Tanaka T, Bobogare A, et al (1994) Prevalence of malaria and its relationship to anemia, blood glucose levels, and serum somatomedin c (IGF-1) levels in the Solomon Islands. Acta Trop 58: 207–220. 7709860 10.1016/0001-706x(94)90015-9

[19] Löfqvist C, Andersson E, Gelander L, Rosberg S, Blum WF, et al (2001) Reference values for IGF-I throughout childhood and adolescence: a model that accounts simultaneously for the effect of gender, age, and puberty. J Clin Endocrinol Metab 86: 5870–5876. 11739455 10.1210/jcem.86.12.8117

[20] Renehan AG, Jones J, O'Dwyer ST, Shalet SM (2003) Determination of IGF-I, IGF-II, IGFBP-2, and IGFBP-3 levels in serum and plasma: comparisons using the Bland–Altman method. Growth Horm IGF Res 13: 341–346. 14624768 10.1016/s1096-6374(03)00112-6

[21] Harada H, Andersen JS, Mann M, Terada N, Korsmeyer SJ (2001) p70S6 kinase signals cell survival as well as growth, inactivating the pro-apoptotic molecule BAD. Proc Natl Acad Sci U S A 98: 9666–9670. 11493700 10.1073/pnas.171301998 PMC55509

[22] Buchon N, Broderick NA, Kuraishi T, Lemaitre B (2010) *Drosophila* EGFR pathway coordinates stem cell proliferation and gut remodeling following infection. BMC Biol 8: 152. 21176204 10.1186/1741-7007-8-152 PMC3022776

[23] Cuervo AM (2008) Autophagy and aging: keeping that old broom working. Trends Genet 24: 604–612. 18992957 10.1016/j.tig.2008.10.002 PMC2745226

[24] Tóth ML, Sigmond T, Borsos É, Barna J, Erdélyi P, et al (2008) Longevity pathways converge on autophagy genes to regulate life span in *Caenorhabditis elegans* . Autophagy 4: 330–338. 18219227 10.4161/auto.5618

[25] Jia K, Thomas C, Akbar M, Sun Q, Adams-Huet B, et al (2009) Autophagy genes protect against *Salmonella typhimurium* infection and mediate insulin signaling-regulated pathogen resistance. Proc Natl Acad Sci U S A 106: 14564–14569. 19667176 10.1073/pnas.0813319106 PMC2731839

[26] Rera M, Azizi MJ, Walker DW (2013) Organ-specific mediation of lifespan extension: More than a *gut* feeling? Ageing Res Rev 12: 436–444. 22706186 10.1016/j.arr.2012.05.003 PMC3498542

[27] Amcheslavsky A, Jiang J, Ip YT (2009) Tissue damage-induced intestinal stem Cell division in *Drosophila* . Cell Stem Cell 4: 49–61. 19128792 10.1016/j.stem.2008.10.016 PMC2659574

[28] Libert S, Chao Y, Zwiener J, Pletcher SD (2008) Realized immune response is enhanced in long-lived *puc* and *chico* mutants but is unaffected by dietary restriction. Mol Immunol 45: 810–817. 17681604 10.1016/j.molimm.2007.06.353

[29] Becker T, Loch G, Beyer M, Zinke I, Aschenbrenner AC, et al (2010) FOXO-dependent regulation of innate immune homeostasis. Nature 463: 369–373. 20090753 10.1038/nature08698

[30] Biteau B, Karpac J, Supoyo S, DeGennaro M, Lehmann R, et al (2010) Lifespan extension by preserving proliferative homeostasis in *Drosophila* . PLoS Genet 6: e1001159. 20976250 10.1371/journal.pgen.1001159 PMC2954830

[31] Luckhart S, Giulivi C, Drexler AL, Antonova-Koch Y, Sakaguchi D, et al (2013) Sustained activation of Akt elicits mitochondrial dysfunction to block *Plasmodium falciparum* infection in the mosquito host. PLoS Pathog 9: e1003180. 23468624 10.1371/journal.ppat.1003180 PMC3585164

[32] Hauck ES, Antonova-Koch Y, Drexler A, Pietri J, Pakpour N, et al (2013) Overexpression of phosphatase and tensin homolog improves fitness and decreases *Plasmodium falciparum* development in *Anopheles stephensi* . Microbes Infect 15: 775–787. 23774695 10.1016/j.micinf.2013.05.006 PMC3788859

[33] Lin Y, Yang Q, Wang X, Liu ZG (2006) The essential role of the death domain kinase receptor-interacting protein in insulin growth factor-I-induced c-Jun N-terminal kinase activation. J Biol Chem 281: 23525–23532. 16793775 10.1074/jbc.M601487200

[34] Meylan E, Tschopp J (2005) The RIP kinases: crucial integrators of cellular stress. Trends Biochem Sci 30: 151–159. 15752987 10.1016/j.tibs.2005.01.003

[35] Georgel P, Naitza S, Kappler C, Ferrandon D, Zachary D, et al (2001) *Drosophila* immune deficiency (IMD) is a death domain protein that activates antibacterial defense and can promote apoptosis. Dev Cell 1: 503–514. 11703941 10.1016/s1534-5807(01)00059-4

[36] Ligoxygakis P (2013) Genetics of immune recognition and response in *Drosophila* host defense. Adv Genet 83: 71–97. 23890212 10.1016/B978-0-12-407675-4.00002-X

[37] Garver LS, Bahia AC, Das S, Souza-Neto JA, Shiao J, et al (2012) *Anopheles* Imd pathway factors and effectors in infection intensity-dependent anti-*Plasmodium* action. PLoS Pathog 8: e1002737. 22685401 10.1371/journal.ppat.1002737 PMC3369948

[38] Garver LS, de Almeida Oliveira G, Barillas-Mury C (2013) The JNK Pathway Is a Key Mediator of *Anopheles gambiae* Antiplasmodial Immunity. PLoS Pathog 9: e1003622. 24039583 10.1371/journal.ppat.1003622 PMC3764222

[39] Akman-Anderson L, Olivier M, Luckhart S (2007) Induction of nitric oxide synthase and activation of signaling proteins in *Anopheles* mosquitoes by the malaria pigment, hemozoin. Infect Immun 75: 4012–4019. 17526741 10.1128/IAI.00645-07 PMC1952000

[40] Delaney JR, Stoven S, Uvell H, Anderson KV, Engstrom Y, et al (2006) Cooperative control of *Drosophila* immune responses by the JNK and NF-kappaB signaling pathways. EMBO J 25: 3068–3077. 16763552 10.1038/sj.emboj.7601182 PMC1500970

[41] Park JM, Brady H, Ruocco MG, Sun H, Williams D, et al (2004) Targeting of TAK1 by the NF-kappa B protein Relish regulates the JNK-mediated immune response in *Drosophila* . Genes Dev 18: 584–594. 15037551 10.1101/gad.1168104 PMC374239

[42] Silverman N, Zhou R, Erlich RL, Hunter M, Bernstein E, et al (2003) Immune activation of NF-kappaB and JNK requires *Drosophila* TAK1. J Biol Chem 278: 48928–48934. 14519762 10.1074/jbc.M304802200

[43] Oliveira Gde A, Lieberman J, Barillas-Mury C (2012) Epithelial nitration by a peroxidase/NOX5 system mediates mosquito antiplasmodial immunity. Science 335: 856–859. 22282475 10.1126/science.1209678 PMC3444286

[44] Figueira TR, Barros MH, Camargo AA, Castilho RF, Ferreira JC, et al (2013) Mitochondria as a source of reactive oxygen and nitrogen species: from molecular mechanisms to human health. Antioxid Redox Signal 18: 2029–2074. 23244576 10.1089/ars.2012.4729

[45] Kuznetsov AV, Kehrer I, Kozlov AV, Haller M, Redl H, et al (2011) Mitochondrial ROS production under cellular stress: comparison of different detection methods. Anal Bioanal Chem 400: 2383–2390. 21336935 10.1007/s00216-011-4764-2

[46] Troncoso R, Vicencio JM, Parra V, Nemchenko A, Kawashima Y, et al (2012) Energy-preserving effects of IGF-1 antagonize starvation-induced cardiac autophagy. Cardiovasc Res 93: 320–329. 22135164 10.1093/cvr/cvr321 PMC3286200

[47] Luckhart S, Vodovotz Y, Cui L, Rosenberg R (1998) The mosquito *Anopheles stephensi* limits malaria parasite development with inducible synthesis of nitric oxide. Proc Natl Acad Sci U S A 95: 5700–5705. 9576947 10.1073/pnas.95.10.5700 PMC20442

[48] Günther C, Neumann H, Neurath MF, Becker C (2013) Apoptosis, necrosis and necroptosis: cell death regulation in the intestinal epithelium. Gut 62: 1062–1071. 22689519 10.1136/gutjnl-2011-301364

[49] Abreu MT, Palladino AA, Arnold ET, Kwon RS, McRoberts JA (2000) Modulation of barrier function during Fas-mediated apoptosis in human intestinal epitelial cells. Gastroenterology 119: 1524–1536. 11113074 10.1053/gast.2000.20232

[50] Chin AC, Teoh DA, Scott KG, Meddings JB, Macnaughton WK, et al (2002) Strain-dependent induction of enterocyte apoptosis by *Giardia lamblia* disrupts epithelial barrier function in a caspase-3-dependent manner. Infect Immun 70: 3673–3680. 12065509 10.1128/IAI.70.7.3673-3680.2002 PMC128105

[51] Pan H, Cai N, Li M, Liu GH, Izpisua Belmonte JC (2013) Autophagic control of cell ‘stemness’. EMBO Mol Med 5: 327–331. 23495139 10.1002/emmm.201201999 PMC3598074

[52] Vessoni AT, Muotri AR, Okamoto OK (2012) Autophagy in stem cell maintenance and differentiation. Stem Cells Dev 21: 513–520. 22066548 10.1089/scd.2011.0526

[53] Yano T, Mita S, Ohmori H, Oshima Y, Fujimoto Y, et al (2008) Autophagic control of listeria through intracellular innate immune recognition in *Drosophila* . Nat Immunol 9: 908–916. 18604211 10.1038/ni.1634 PMC2562576

[54] Randow F, Münz C (2012) Autophagy in the regulation of pathogen replication and adaptive immunity. Trends Immunol 33: 475–487. 22796170 10.1016/j.it.2012.06.003 PMC3461100

[55] Benjamin JL, Sumpter R Jr, Levine B, Hooper LV (2013) Intestinal epithelial autophagy is essential for host defense against invasive bacteria. Cell Host Microbe 13: 723–734. 23768496 10.1016/j.chom.2013.05.004 PMC3755484

[56] Randall-Demllo S, Chieppa M, Eri R (2013) Intestinal epithelium and autophagy: partners in gut homeostasis. Front Immunol 4: 301. 24137160 10.3389/fimmu.2013.00301 PMC3786390

[57] Micchelli CA, Perrimon N (2005) Evidence that stem cells reside in the adult *Drosophila* midgut epithelium. Nature 439: 475–479. 16340959 10.1038/nature04371

[58] Ohlstein B, Spradling A (2005) The adult *Drosophila* posterior midgut is maintained by pluripotent stem cells. Nature 439: 470–474. 16340960 10.1038/nature04333

[59] Suzuki K, Ohsumi Y (2007) Molecular machinery of autophagosome formation in yeast *Saccharomyces cerevisiae* . FEBS Lett 581: 2156–2161. 17382324 10.1016/j.febslet.2007.01.096

[60] Oh SW, Mukhopadhyay A, Svrzikapa N, Jiang F, Davis RJ, et al (2005) JNK regulates lifespan in *Caenorhabditis elegans* by modulating nuclear translocation of forkhead transcription factor/DAF-16. Proc Natl Acad Sci U S A 102: 4494–4499. 15767565 10.1073/pnas.0500749102 PMC555525

[61] Wang MC, Bohmann D, Jasper H (2005) JNK extends life span and limits growth by antagonizing cellular and organism-wide responses to insulin signaling. Cell 121: 115–125. 15820683 10.1016/j.cell.2005.02.030

[62] Biteau B, Hochmuth CE, Jasper H (2008) JNK activity in somatic stem cells causes loss of tissue homeostasis in the aging *Drosophila* gut. Cell Stem Cell 3: 442–455. 18940735 10.1016/j.stem.2008.07.024 PMC3225008

[63] Owusu-Ansah E, Song W, Perrimon N (2013) Muscle mitohormesis promotes longevity via systemic repression of insulin signaling. Cell 155: 699–712. 24243023 10.1016/j.cell.2013.09.021 PMC3856681

[64] Wen Z, Gulia M, Clark KD, Dhara A, Crim JW, et al (2010) Two insulin-like peptide family members from the mosquito *Aedes aegypti* exhibit differential biological and receptor binding activities. Molec Cell Endocrinol 328: 47–55. 20643184 10.1016/j.mce.2010.07.003 PMC2957182

[65] Rasband WS, ImageJ (1997) U. S. National Institutes of Health, Bethesda, Maryland, USA, http://imagej.nih.gov/ij/, 1997–2012.

