# Supplementary figures and images for: Human IGF1 Regulates Midgut Oxidative Stress and Epithelial Homeostasis to Balance Lifespan and Plasmodium falciparum resistance in Anopheles stephensi
Source: PLoS Pathog. 2014 Jun 26;10(6):e1004231. doi: 10.1371/journal.ppat.1004231 (PMC4072789; doi:10.1371/journal.ppat.1004231)

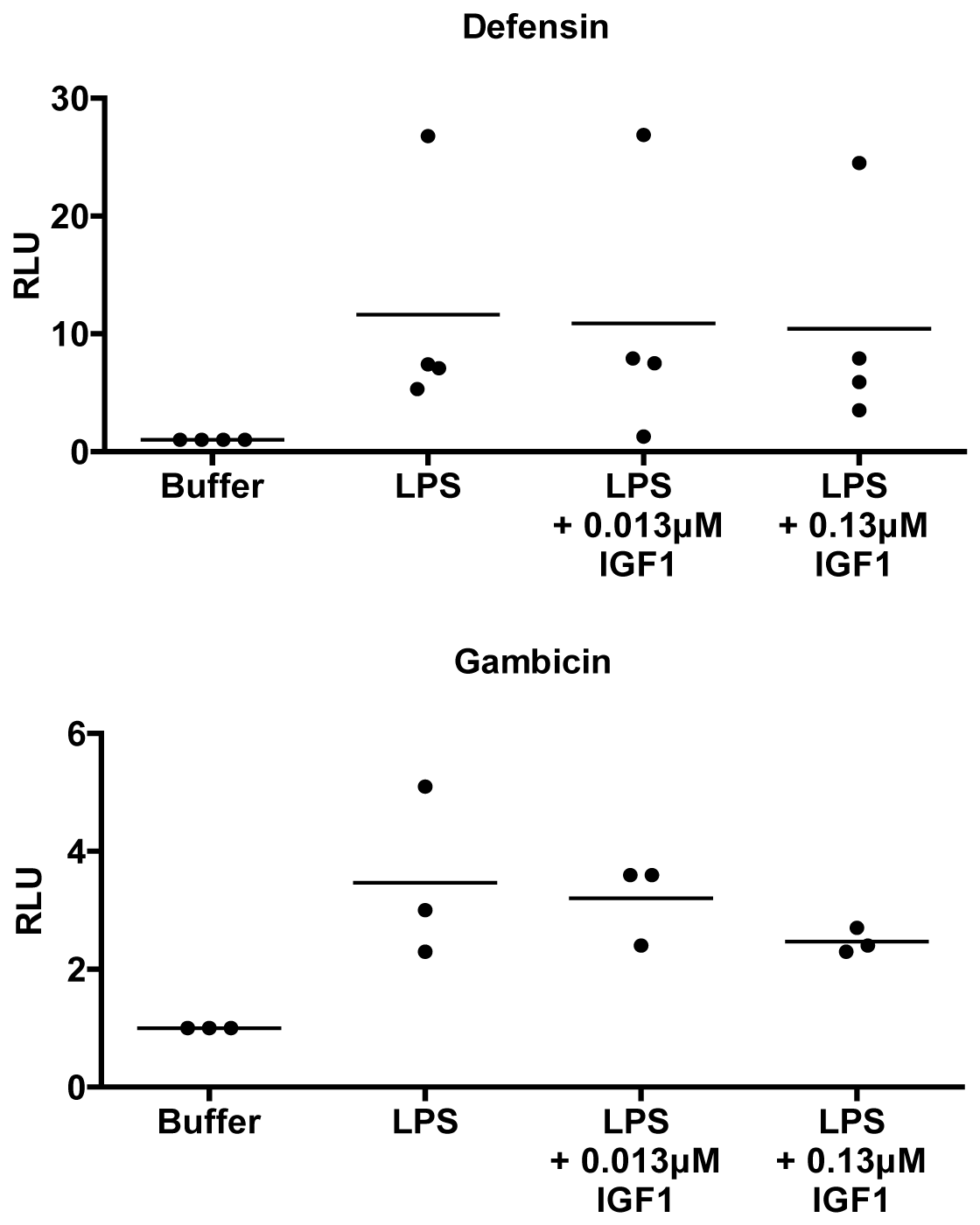

Supplement: Figure S1 — Raw (non-normalized) data from Figure 2A . Human IGF1 did not alter antimicrobial peptide (AMP) promoter activity in immune-activated ASE cells. Graphs depicts relative light units (RLU) from promoter-reporter assays in transfected cells stimulated with or without LPS and with or without IGF1. (TIF) [file ppat.1004231.s001.tif]

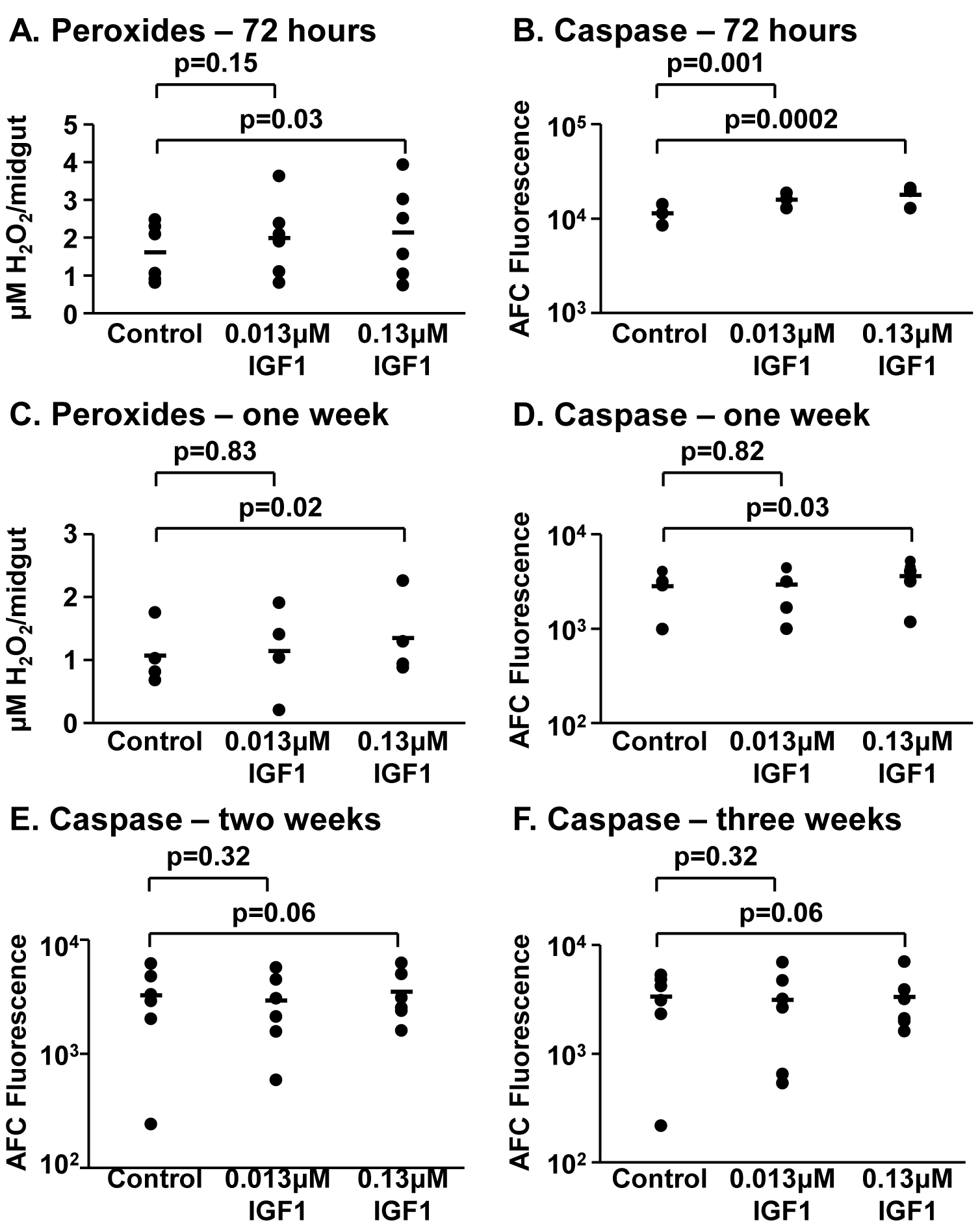

Supplement: Figure S2 — Raw (non-normalized) data from Figure 6 . Peroxide levels in midguts at 72 h (A) and one week (C) following IGF1 treatment. Peroxides were quantified in pools of five midguts; three pools were collected per treatment per experiment. Experiments were replicated three times with separate cohorts of mosquitoes. Graphs show the µM H2O2/midgut. Midgut caspase-3 activity at 72 h (B), one week (D), two weeks (E), and three weeks (F) following IGF1 treatment. Graphs show AFC fluorescence (caspase-3 activity). Three pools of five midguts per treatment were collected for 72 h and one week timepoints and 1–2 pools were collected for two and three week timepoints. Experiments were replicated 4–6 times with separate cohorts of mosquitoes. (TIF) [file ppat.1004231.s002.tif]
